# Examining factors associated with experiencing cardiac arrhythmias in Black and White breast cancer survivors who received anthracyclines or trastuzumab

**DOI:** 10.1007/s10549-025-07671-0

**Published:** 2025-03-27

**Authors:** Arnethea L. Sutton, Jinlei Zhao, Jian He, Katherine Y. Tossas, Wendy Bottinor, Vanessa B. Sheppard

**Affiliations:** 1 Department of Kinesiology and Health Sciences, Virginia Commonwealth University, P.O. Box 843021, Richmond, VA 23220 USA; 2 VCU Massey Comprehensive Cancer Center, Richmond, VA USA; 3 Department of Social and Behavioral Sciences, Virginia Commonwealth University School of Public Health, Richmond, VA USA; 4 Division of Cardiology, Department of Internal Medicine, Virginia Commonwealth University School of Medicine, Richmond, VA USA

**Keywords:** Breast cancer, Racial disparities, Cardiac arrhythmias, Hypertension

## Abstract

**Purpose:**

Racial disparities exist regarding cardiovascular (CV) toxicities following breast cancer treatment; however, studies on racial differences in cardiac arrhythmias are lacking. This study examined associations between demographic and clinical factors and arrhythmia diagnosis in Black and White breast cancer survivors.

**Methods:**

This study included a retrospective cohort of Black and White women who were diagnosed with breast cancer and who received potentially cardiotoxic treatment. Cardiac arrhythmia data were captured via International Classification of Diseases, Tenth and Ninth Versions (ICD-10 and ICD-9). Experiences with cardiac arrhythmias were compared across racial groups. The associations of demographic and clinical factors with cardiac arrhythmias were evaluated using logistic regression for all women and in race-stratified models.

**Results:**

Thirty-three percent of the total 860 women in our study sample (mean (standard deviation) age 50.3 (10.7) years) old) experienced cardiac arrhythmias. In bivariate analyses, we observed a statistically discernible association between race and arrhythmia status following a breast cancer diagnosis (p = 0.004); however, this association was no longer significant in the multivariable model. In race-stratified multivariable analysis, the odds of experiencing arrhythmias in Black women over 50 years old are 51% lower than in Black women aged 50 years old or younger (adjusted odds ratio (OR): 0.49; 95% confidence interval (CI): 0.28, 0.86). All else being equal, Black women with hypertension had 2.68 times (95% CI: 1.51, 4.81) higher odds of experiencing arrhythmias than those without hypertension. White women with obesity had higher odds of experiencing arrhythmias than those with normal weight or underweight status. (adjusted OR 1.93: [1.17, 3.20]).

**Conclusion:**

Survivors with chronic conditions like hypertension and obesity may require enhanced cardiac surveillance. Further investigation into hypertension management in Black survivors may shed light on its impact on CV toxicities in this group.

## Introduction

Cardiac arrhythmias, inclusive of atrial fibrillation and conduction disorders are of growing concern for women who receive potentially cardio-toxic therapy (e.g., anthracycline chemotherapies, human epidermal receptor 2 (HER2) targeted therapy) for breast cancer [1–3]. Concerns are valid as studies show that women with breast cancer are more likely to experience cardiovascular disease when compared to women without breast cancer, and older women with breast cancer are more likely to succumb to cardiovascular disease than to their breast cancer [4–7].

Cardiac arrhythmias, inclusive of diagnoses such as atrial fibrillation and conduction disorders, are one example of CV toxicities that remain understudied in women with breast cancer. While women with arrhythmias may experience symptoms, such as palpitations or dizziness, others may experience no symptoms as arrhythmias are sometimes coined as ‘silent’ [8]. More importantly, although the causative relationships are not fully understood, arrhythmias may lead to cardiomyopathy, heart failure, or even sudden cardiac death [9–11]. At present, few studies have examined the relationship between race and risk factors for cardiovascular disease and the development of arrhythmias upon receipt of potentially cardiotoxic treatment for breast cancer.

Although all women who receive cardiotoxic treatment are at-risk of CV toxicities, studies have reported on racial disparities in CV toxicities following breast cancer treatment. Black women are up to three times more likely to experience CV toxicities when compared to White women [12]. Reasons for this disparity are not clearly understood. Studies that examine racial differences in CV toxicities report that even after accounting for CV disease risk factors, such as hypertension and diabetes, and socioeconomic factors, the disparity persists [13, 14]. Most studies that examine racial differences in CV toxicities focus on heart failure and left ventricular dysfunction, highlighting a knowledge gap in potential disparities in cardiac arrhythmias.

The aims of this study were to examine racial differences for cardiac arrhythmias following a breast cancer diagnosis, assess the contribution of demographic and clinical factors, and comorbid conditions on an arrhythmia diagnosis, and to examine whether factors associated with arrhythmias differ by race. We hypothesized that Black women will be more likely to experience arrhythmias when compared to White women and that the contribution of clinical factors on experiences with arrhythmias will differ by race.

## Methods

### Study design and participants

Using electronic health record data from Virginia Commonwealth University (VCU) Health System and the VCU Massey Comprehensive Cancer Center, we evaluated women diagnosed with American Joint Committee on Cancer (AJCC) stages I-III or unstaged invasive breast cancer who received an anthracycline-based chemotherapy and/or trastuzumab between 2009 and 2019. Women who self-identified as Black or White were included. We excluded women who had cardiovascular disease prior to breast cancer diagnosis. Our outcome of interest was cardiac arrhythmias. This study was approved by the VCU institutional review board.

To guide the abstraction of appropriate International Classification of Disease (ICD)−9 and 10 codes pertaining to arrhythmias, we consulted the International Cardio-Oncology Society consensus statement that defines CV toxicities including arrhythmias [15]. Diagnoses included, but are not limited to ventricular or atrial fibrillation, atrial flutter, tachycardia, and conduction disorders, including atrioventricular block.

Demographic factors included race, age at diagnosis, marital status (married vs. unmarried), insurance (i.e., government-issued, private), and geography (metro vs. non-metro). Clinical factors comprised AJCC stage, endocrine receptor, progesterone receptor, and human epidermal growth factor 2 status, and the type of cardiotoxic medication women received (i.e., anthracycline-based chemotherapy, trastuzumab, or both). Surgery type included mastectomy, lumpectomy, and re-excision of the biopsy site for gross or microscopic residual disease, classified as ‘other’. Additionally, comorbid conditions known to contribute to CV toxicities, such as hypertension and diabetes, were determined via ICD-9 or 10 codes while body mass index (BMI) at diagnosis was abstracted from the medical record.

### Statistical analysis

We computed descriptive statistics, including means ± standard deviations and frequencies (percentages), for all demographic and clinical factors for all women in our study sample, and also stratified by race and race and cardiac arrhythmia status following breast cancer diagnosis. In bivariate analyses, we assessed associations of race and arrhythmia status following breast cancer diagnosis with each demographic and clinical factor, using two-sided chi square tests of independence and t tests for qualitative and quantitative variables, respectively. Variables that were significantly associated with arrhythmia status at the significance level of 0.05 were included in the multivariable logistic regression models, and they are age at diagnosis, cancer stage, surgery type, BMI, diabetes status, hypertension status, trastuzumab use, and anthracycline chemotherapy use. SAS 9.4 was the main analysis tool [16].

## Results

Our study sample comprised 860 women. Most of them were White (62%), married (55%), received a mastectomy (80%), and received anthracycline-based chemotherapy (78%) (Table 1). Compared to White women, Black women were younger (48 years old vs. 52 years old) at diagnosis, more likely to be unmarried (66.3% vs. 31.6%), and had higher prevalences of obesity (60.7% vs. 30.5%), hypertension (26.1%, vs. 6.2%), and diabetes (13.5% vs. 3.6%).

**Table 1:** Demographic and clinical characteristics of Black and White breast cancer survivors

Participant Characteristics	Total N = 860	Black (N = 326, 37.9%)	White (N = 534, 62.1%)	p-value
Age, years (mean ± SD)	50.3 ± 10.7	48.4 ± 11.0	51.5 ± 10.4	<0.001†
Age Group, n (%) ≤ 50 > 50	439 (51.0) 421 (49.0)	188 (57.7) 138 (42.3)	251 (47.0) 283 (53.0)	0.002
Marital Status, n (%) Married Unmarried	475 (55.2) 385 (44.8)	110 (33.7) 216 (66.3)	365 (68.4) 169 (31.6)	<0.001
Insurance Status, n (%) Government-Issued Private Uninsured Self-pay Missing	163 (19.0) 512 (59.5) 97 (11.3) 84 (9.8) 4 (0.5)	72 (22.1) 158 (48.5) 51 (15.6) 41 (12.6) 4 (1.2)	91 (17.0) 354 (66.3) 46 (8.6) 43 (8.1)	<0.001
Geography, n (%) Metro Non-metro Missing	737 (85.7) 120 (14.0) 3 (0.3)	273 (83.7) 51 (15.6) 2 (0.6)	464 (87.0) 69 (12.9) 1 (0.2)	0.25
BMI, n (%) Underweight/Normal Weight Overweight Obesity	229 (26.6) 270 (31.4) 361 (42.0)	36 (11.0) 92 (28.2) 198 (60.7)	193 (36.1) 178 (33.3) 163 (30.5)	<0.001
Stage, n (%) I II III NA	176 (20.5) 362 (42.1) 152 (17.7) 170 (19.8)	59 (18.1) 142 (43.6) 66 (20.2) 59 (18.1)	117 (21.9) 220 (41.2) 86 (16.1) 111 (20.8)	0.18
ER Status, n (%) Negative Positive Unknown	377 (43.8) 468 (54.4) 15 (1.7)	184 (56.4) 136 (41.7) 6 (1.8)	193 (36.1) 332 (62.2) 9 (1.7)	<0.001
PR Status, n (%) Negative Positive Unknown	461 (53.6) 386 (44.9) 13 (1.5)	216 (66.3) 107 (32.8) 3 (0.9)	245 (45.9) 279 (52.2) 10 (1.9)	<0.001
HER2 Status, n (%) Negative Positive Unknown	286 (33.3) 204 (23.7) 370 (43.0)	140 (42.9) 78 (23.9) 108 (33.1)	146 (27.3) 126 (23.6) 262(49.1)	0.02
Surgery Type, n (%) Lumpectomy or Excisional Biopsy Mastectomy Other Missing	103 (12.0) 691 (80.3) 34 (4.0) 32 (3.7)	25 (7.7) 273 (83.7) 9 (2.8) 19 (5.8)	78 (14.6) 418 (78.3) 25 (4.7) 13 (2.4)	0.005
Anthracycline-based Chemotherapy, n (%) No Yes	190 (22.1) 670 (77.9)	64 (19.6) 262 (80.4)	126 (23.6) 408 (76.4)	0.17
Trastuzumab, n (%) No Yes	561 (65.2) 299 (34.8)	225 (69.0) 101 (31.0)	336 (62.9) 198 (37.1)	0.07
Diabetes, n (%) No Yes	797 (92.7) 63 (7.3)	282 (86.5) 44 (13.5)	515 (96.4) 19 (3.6)	<0.001
Hypertension, n (%) No Yes	742 (86.2) 118 (13.8)	241 (73.9) 85 (26.1)	501 (93.8) 33 (6.2)	<0.001
Arrhythmias, n (%) No Yes	573 (66.6) 287 (33.4)	198 (60.7) 128 (39.3)	375 (70.2) 159 (29.8)	0.004

^†^P-value from the two-sample unpaired t-test under the equal variance assumption

Most women in our sample did not experience arrhythmias during or following treatment (67%). Black women had a higher prevalence of arrhythmias than White women (39.3% vs. 29.8%) (Table 2). Additionally, there were statistically discernible associations of cardiac arrhythmias with body mass index classification (p < 0.001) and breast cancer stage (p = 0.01). We did not observe a significant association between arrhythmias and age at the diagnosis of breast cancer (p = 0.15) (see Table 2).

**Table 2:** Demographic and clinical factors of Black and White breast cancer survivors by arrhythmia diagnosis status

Participant characteristics	Experienced arrhythmias		p-value
	No (N = 573, 66.6%)	Yes (N = 287, 33.4%)	
Age, years (mean ± SD)	50.7 ± 10.5	49.8 ± 11.1	0.15†
Age Group, n (%) ≤ 50 > 50	285 (49.7) 288 (50.3)	154 (53.7) 133 (46.3)	0.28
Race, n (%) Black White	198 (34.6) 375 (65.4)	128 (44.6) 159 (55.4)	0.004
Marital Status, n (%) Married Unmarried	329 (57.4) 244 (42.6)	146 (50.9) 141 (49.1)	0.07
Insurance Status, n (%) Government-Issued Private Uninsured Self-pay Missing	103 (18.0) 355 (62.0) 62 (10.8) 51 (8.9) 2 (0.3)	60 (20.9) 157 (54.7) 35 (12.2) 33 (11.5) 2 (0.7)	0.24
Geography, n (%) Metro Non-metro Missing	494 (86.2) 76 (13.3) 3 (0.5)	243 (84.7) 44 (15.3) 0 (0.0)	0.43
BMI, n (%) Underweight/Normal Weight Overweight Obesity	166 (29.0) 193 (33.7) 214 (37.3)	63 (22.0) 77 (26.8) 147 (51.2)	< 0.001
Stage, n (%) I II III NA	127 (22.2) 241 (42.1) 86 (15.0) 119 (20.8)	49 (17.1) 121 (42.2) 66 (23.0) 51 (17.8)	0.01
ER Status, n (%) Negative Positive Unknown	248 (44.3) 316 (55.1) 9 (1.6)	129 (44.9) 152 (53.0) 6 (2.1)	0.59
PR Status, n (%) Negative Positive Unknown	305 (53.2) 260 (45.4) 8 (1.4)	156 (54.4) 126 (43.9) 5 (1.7)	0.64
HER2 Status, n (%) Negative Positive Unknown	178 (31.1) 133 (23.2) 262 (45.7)	108 (37.6) 71 (24.7) 108 (37.6)	0.50
Surgery Type, n (%) Lumpectomy or Excisional Biopsy Mastectomy Other Missing	81 (14.1) 450 (78.5) 27 (4.7) 15 (2.6)	22 (7.7) 241 (84.0) 7 (2.4) 17 (5.9)	0.008
Anthracycline-based Chemotherapy, n (%) No Yes	128 (22.3) 445 (77.7)	62 (21.6) 225 (78.4)	0.81
Trastuzumab, n (%) No Yes	379 (66.1) 194 (33.9)	182 (63.4) 105 (36.6)	0.43
Diabetes, n (%) No Yes	540 (94.2) 33 (5.8)	257 (89.5) 30 (10.5)	0.01
Hypertension, n (%) No Yes	518 (90.4) 55 (9.6)	224 (78.0) 63 (22.0)	<0.001

^†^P-value from the two-sample unpaired t-test under the equal variance assumption

 In the multivariable logistic regression model with all women in our sample, race was no longer significantly associated with arrhythmia status following breast cancer diagnosis (adjusted OR: 1.02 [95% Confidence Interval (CI): 0.72, 1.43]) (Table 3), after adjusting for age, cancer stage, surgery type, BMI, diabetes status, hypertension status, trastuzumab use, and anthracycline chemotherapy use. Controlling for the same set of covariates as for the model with all women, race-stratified multivariable logistic regression models revealed different patterns of associations with arrhythmia status between Black and White women. Age was significantly associated with arrhythmia status only among Black women, and the odds of experiencing arrhythmias in Black women over 50 years old are 51% lower than in Black women aged 50 years old or younger (adjusted OR: 0.49;95% CI: 0.28, 0.86) (Table 3). Hypertension status was only significantly associated with arrhythmia status among  Black women. All else being equal, Black women with hypertension had 2.68 times (95% CI: 1.51, 4.81) higher odds of experiencing arrhythmias than those without hypertension. Additionally, only among White women, obesity was associated with increased odds of experiencing arrhythmias compared to normal weight or underweight status (adjusted OR:1.93; 95% CI: 1.17, 3.20). However, there was not a significant association in experiencing arrhythmias between White women with overweight and those with normal/underweight (adjusted OR: 1.89; 95% CI: 0.86, 4.14).

**Table 3:** Adjusted odds ratios of experience with cardiac arrhythmias in all women and by race

Characteristics	All women (n = 860) OR (95% CI)	Black women (n = 326) OR (95% CI)	White women (n = 534) OR (95% CI)
Race Black White	1.02 (0.72, 1.43) Reference	–	–
Age Group > 50 ≤ 50	0.77 (0.56, 1.06) Reference	0.49 (0.28, 0.86)* Reference	1.03 (0.69, 1.54) Reference
Stage II III Unknown I	1.16 (0.75, 1.81) 1.83 (1.09, 3.07)* 0.93 (0.55, 1.59) Reference	1.99 (0.95, 4.33) 3.24 (1.39, 7.84)** 2.25 (0.92, 5.60) Reference	0.81 (0.47, 1.43) 1.29 (0.66, 2.52) 0.49 (0.24, 0.97)* Reference
Trastuzumab Yes No	1.60 (1.03, 2.49)* Reference	1.77 (0.83, 3.73) Reference	1.41 (0.79, 2.47) Reference
Anthracycline-based Chemotherapy Yes No	1.37 (0.80, 2.35) Reference	0.76 (0.30, 1.89) Reference	1.87 (0.93, 3.79) Reference
Surgery Type Lumpectomy or excisional biopsy Other Mastectomy	0.56 (0.33, 0.92)* 0.63(0.25, 1.42) Reference	0.73 (0.27, 1.83) 2.04(0.46, 8.55) Reference	0.44 (0.22, 0.84)* 0.32 (0.07, 0.96) Reference
BMI Obesity Overweight Normal/Underweight	1.54 (1.02, 2.33)* 1.03 (0.68, 1.58) Reference	0.88 (0.38, 2.05) 0.59 (0.24, 1.47) Reference	1.93 (1.17, 3.20)* 1.10 (0.67, 1.82) Reference
Diabetes Yes No	1.43 (0.79, 2.55) Reference	1.54 (0.72, 3.26) Reference	2.02 (0.71, 5.79) Reference
Hypertension Yes No	2.30 (1.47, 3.58)*** Reference	2.68 (1.51, 4.81)*** Reference	1.89 (0.86, 4.14) Reference

^*^p < 0.05

^**^p < 0.01

^***^p < 0.001

## Discussion

This is one of the first studies that characterize racial differences in factors associated with cardiac arrhythmias in breast cancer survivors. Although experiencing arrhythmias were significantly associated with race in bivariate analyses, after controlling for covariates (e.g., age, BMI classification, treatment), this association was no longer significant. In addition, this study highlights the importance of clinical targets, specifically chronic conditions that are risk factors for CV toxicity, that may improve outcomes in women. Our results suggest that chronic comorbid conditions, namely hypertension, diabetes, obesity not only contribute to arrhythmias, but they may also play a role in interracial differences in experiences with cardiac arrhythmias. Hypertension was significantly associated with cardiac arrhythmias amongst Black women only while the same was true regarding obesity amongst White women only. This finding, to our knowledge, has not been previously reported.

Approximately 33% of women experienced cardiac arrhythmias following a breast cancer diagnosis in this study. Proper management of cardiac arrhythmias during and following treatment for breast cancer is critical during survivorship, particularly as studies show that arrhythmias may lead to additional cardiac complications, such as ischemic stroke, tachycardia-induced cardiomyopathy, and heart failure [17–20]. A recent study that utilized the Surveillance, Epidemiology, and End-Results-Medicare-Linked database to investigate atrial fibrillation in women with breast cancer reported an association between increased 1-year cardiovascular mortality in women with an atrial fibrillation diagnosis following a breast cancer diagnosis [20]. In addition to the need for enhanced surveillance, future research is needed to assess the onset of additional CV toxicities and CV-related mortality associated with arrhythmias following a breast cancer diagnosis.

In our study, we found that after controlling for covariates of interest, there was no differential association in the development of arrhythmias when comparing Black and White women. Though not specifically focused on arrhythmias, in studies of women with breast cancer, most find that Black women are more likely to experience CV toxicities than White women, even after controlling for plausible factors related to CV toxicities (e.g., cardiotoxic treatment, hypertension) [13, 21]. Conversely, in other studies, after accounting for the aforementioned factors, racial differences were no longer significant [22]. Additionally, mixed findings amongst women with cancer prompt a need to understand the roles of treatment modalities (i.e., anthracycline-based chemotherapies, trastuzumab, radiation) and individual CV toxicities mechanisms on racial disparities in CV toxicities.

Race stratified analyses revealed that younger Black women were more likely to experience arrhythmias than older Black women. This is a novel finding as older age remains a risk factor for arrhythmias and other CV toxicities, in general. One possible explanation for this finding involves adherence to anti-hypertensive medications and subpar hypertension management. A study of Black women who were prescribed antihypertensive medications found that while women of all groups had poor adherence, the highest nonadherence was amongst younger Black women, between ages 40–49 [23]. Future work is needed that centers young Black breast cancer survivors and seeks to understand multilevel contributors (e.g., stress, cancer, and hypertension care delivery) that may contribute to their risk of cardiac arrhythmias. This is especially salient as a recent study by Tanake et al. reported an increase in cardiovascular deaths related to atrial fibrillation among younger adults [24].

Comorbid conditions, namely those that are risk factors for CV toxicities, were drivers of arrhythmias in Black and White women. Patterns, however, varied by race. Amongst Black women only, hypertension was significantly associated with arrhythmias. As mentioned for younger Black women, this may be due in part to nonadherence to antihypertensive medication and inadequate hypertension control. A study by Hershman et al. reported that Black breast cancer survivors were more likely to be nonadherent when compared to breast cancer survivors of other races and ethnicities. Additionally, nonadherence was associated with a greater risk of cardiac events following a breast cancer diagnosis [25]. It is also salient to consider guidelines for treating hypertension in Black women. The most recent clinical practice guidelines recommend two or more anti-hypertensive medications to achieve a blood pressure of < 130/80 mm HG, “especially in African American adults [26].” Obesity, a known risk factor for CV toxicities, was only significant for White women. These findings not only highlight a need for increased cardiac surveillance in women with comorbid conditions but, with regard to BMI and obesity, there may be better measures, such as waist circumference or visceral fat measurements, that may provide more accurate risk predictions for arrhythmias and other CV toxicities.

We acknowledge that this study has limitations. Time to event analysis would have provided more insight regarding survivors’ risk of arrhythmias and more details regarding the occurrence of an event during a specified follow-up period. We were unable to include smoking, coronary artery disease, and radiation, known risk factors of CV toxicities, in the analyses, as these data were not available. In addition, the sample for this study was from one healthcare system, limiting generalizability to other patient populations. Despite the limitations, there are noted strengths to report. This study included a high representation of Black breast cancer survivors, when compared to similar studies, allowing for race-stratified analysis. While some risk factors were excluded, this study did include BMI, a factor that has not been fully explored with regard to racial differences in CV toxicities. This study also included more age and payer-type diversity, unlike the other studies that mostly report on women administrative databases who are 66 years of age and older who have Medicare only.

Compared to older Black women, younger Black women were more likely to experience arrhythmias. Additionally, the differential impact of comorbid conditions (i.e., obesity, hypertension) on experiences with cardiac arrhythmias supports further research on behavioral factors and biological mechanisms that may inform enhanced surveillance and cardiac management for Black and White breast cancer survivors who receive cardiotoxic treatments. These approaches may ultimately reduce racial disparities in breast cancer morbidity and mortality.

## Data Availability

Data archiving is not mandated but will be made available on reasonable request.
